# Fetal mortality at and beyond term in singleton pregnancies in Baden-Wuerttemberg/Germany 2004–2009

**DOI:** 10.1007/s00404-013-2957-y

**Published:** 2013-07-10

**Authors:** Erich Weiss, Kerstin Krombholz, Martin Eichner

**Affiliations:** 1 Department of Obstetrics and Gynecology, Perinatal Centre Kliniken Boeblingen, Kliniken Boeblingen, Teaching Hospital of Tuebingen University Medical School, Bunsenstrasse 120, Boeblingen, 71032 Germany; 2 Institute for Clinical Epidemiology and Applied Biometry, Eberhard Karls University, Tübingen, Germany

**Keywords:** Postterm pregnancy, Postdates, Perinatal mortality, Stillbirth, Intrauterine fetal death

## Abstract

**Objective:**

To evaluate the risk of intrauterine fetal death (IUFD) in low-risk pregnancies at and beyond term under conditions of fetal monitoring practiced in Baden-Wuerttemberg/Germany (BW).

**Methods:**

We performed a retrospective analysis of 472,843 low-risk singleton pregnancies in BW, using data from the local National Medical Birth registry. The setting of fetal monitoring was uniform during the analyzed time period (2004–2009). We calculated the IUFD rate per 1,000 ongoing pregnancies for each gestational week and compared our results to other published studies using the same calculation scheme.

**Results:**

Our study demonstrates a markedly lower risk of IUFD between 37+0/7 and 42+6/7 weeks of pregnancy when compared with data from Scotland, England, and Sweden collected between 1985 and 1996. When our data were compared to a recently published study from California reporting on deliveries between 1997 and 2006, the risk for IUFD was only significantly lower from 41 weeks onward. The distribution of weekly delivery rates shows a trend to earlier deliveries in weeks 37+0/7 to 39+6/7 for the actual cohorts from California and BW.

**Conclusion:**

In our study, the risk for IUFD in pregnancies going beyond term is remarkably lower than found in studies published about other countries. Our results do not support current guidelines which recommend a routine induction of labor in low risk pregnancies at 41+0/7 weeks of pregnancy.

## Introduction

Dated from the first day of the last menstruation period (LMP), the duration of a normal pregnancy is 280 days of gestational age or 40+0/7 weeks of pregnancy (WOP). Term pregnancy is defined as a gestation lasting between 37+0/7 WOP and 41+6/7 WOP. Pregnancies going beyond 41+6/7 WOP are defined as postterm pregnancies (WHO definition).The reported rate of postterm pregnancy (>41+6/7 WOP) published in the literature ranges from 4 to 14 % [1–3], but accurate pregnancy dating by routine first trimester ultrasound reduces this by eliminating dating errors [4]. In Germany, less than 0.9 % of all pregnancies are postterm [5]. Far more common, and therefore of significant interest, are pregnancies lasting beyond the estimated due date of 40+0/7 WOP since this concerns 33–48 % of all pregnancies.

Until the late 1990s of the last century, scientific interest had focused on postterm pregnancies (≥42+0/7 weeks of gestation). Many retrospective trials demonstrated increased perinatal and first-year mortality as well as increased newborn morbidity due to aspiration, umbilical cord complications, perinatal asphyxia, pneumonia, sepsis, neurological morbidity, peripheral nerve damage, and fractures [3, 6–8]. This resulted in the recommendation, found in many national guidelines, to induce labor at a confirmed gestational age of 42+0/7 weeks [9–12]. However, risks of perinatal morbidity and mortality for the fetus are also present before a gestational age of 42+0/7 weeks. Several retrospective trials demonstrated that the risk of fetal morbidity and mortality already steadily rises starting from the 39th WOP [7, 13–18].

Many epidemiological studies reported a low risk of intrauterine fetal death (IUFD) for 37–42 WOP and only a minor rise in stillbirths after the 42nd WOP. This is due to the fact that the authors calculated the IUFD rate as the number of stillbirths per 1,000 births for each WOP. However, correct calculation of this risk requires that the number of IUFDs is divided by the number of subjects at risk for IUFD [18], i.e. it must not be based on the babies who are born in a given week, but rather on all fetuses who are alive at the beginning of that WOP. Yudkin [19] was the first to point out this correct calculation in 1987. The retrospective analyses based on the correct calculation reported a risk of stillbirth rising from 0.35 to 0.4 per 1,000 “ongoing” pregnancies in the 37th WOP to 2.1–3.1 per 1,000 in the 43rd WOP, i.e., a rise by a factor of 6–7 [7, 17–20].

These aspects favor the initiation of a prospective randomized trial aimed at answering the following question: “Do fetal morbidity and mortality benefit from terminating pregnancy before 42+0/7 weeks of gestation?” Since IUFD is such a rare event, one would have to recruit about 150,000 pregnant women with low-risk pregnancy at 40–42 WOP [21]. However, due to ethical and legal aspects, such a study would never be accepted in any country. Meta-analyses with rather small prospective trials demonstrated that routine induction of labor at 41+0/7 WOP could reduce fetal morbidity and mortality without increasing the rate of Cesarean delivery [15, 22, 23]. In these meta-analyses, only four of 14 studies could estimate the IUFD risk. As three of these four trials dated from 1969 to 1992 [22] and as they were dominated by one large study [24], the evidence from these meta-analyses is limited.

The present study is a retrospective analysis of the IUFD risk between 37-0/7 and 42+6/7 WOP under pregnancy care as practiced in Baden-Wuerttemberg (BW), Germany. The results with respect to the risk of IUFD at each gestational week will be compared to other international retrospective studies.

## Materials and methods

### Data sources

Our paper is based on data provided by the Office of Quality Assurance in Hospitals (GeQiK) in Stuttgart, Germany, comprising 180 parameters for every birth in BW (Article 137 Sect. 1 of Volume V of the Social Security Code makes it mandatory for all hospitals in Germany to collect these data and submit them to the GeQiK). The data set provided to us covers all births in BW hospitals between 2004 and 2009.

### Methods

The data set for this study, describing the period from 2004 to 2009, was obtained from the local National Medical Birth registry which collects detailed data on all hospital deliveries in BW since 1979. The data provided to us were administered on a password protected computer at the GeQiK and were anonymized according to current data privacy laws and regulations. For reasons of data privacy, the parameters had to be analyzed on site at GeQiK, using the JMP software by the SAS Institute.

We obtained institutional review board approval from the steering committee of the GeQik and from the ethics committee at the Medical School of the University Tübingen. Because the data were deidentified and are part of the National Birth registry, informed consent was not required.

Statistical calculations including relative risks and 95 % confidence intervals (CI) were performed using Excel and JMP software. To calculate *p* values, Fisher’s exact test (two sided) was used.

All pregnancies with either pre- or postnatally diagnosed fetal malformations, multiple pregnancies, and all data without confirmed gestational age (missing ultrasound study before the 20th week) were excluded from the analysis.

The risk of intrauterine fetal death in a given week of pregnancy was calculated as follows: (number of stillbirths)/(number of ongoing pregnancies at the beginning of the week of gestation).

The results are compared with the outcomes in similar retrospective studies from Scotland [18], England [7], Sweden [20], and California (US) [25]. For each trial, the risk for IUFD was calculated in identical fashion, based on the number of ongoing pregnancies. Between 2004 and 2009, all hospitals in BW participating in the Birth Registry started fetal monitoring (non-stress test) from 40+0/7 WOP onwards (2-day intervals).

## Results

According to the data of the Federal Statistical Office of Germany, 559,148 newborns were registered in Baden-Wuerttemberg (BW) between 2004 and 2009 [26]; 553,199 were born in hospitals.

The GeQiK data represent 99.4 % of the 553,199 babies born in BW hospitals. After having excluded premature births (<37+0/7 WOP), multiple pregnancies, malformations, and all cases without assessment of the gestational age by ultrasound < 20 WOP, 472,843 data sets were available for analysis.

Table 1 reports the distribution of deliveries between 37+0/7 and 42+6/7 WOP. In BW, 99 % of all children are born before 42+0/7 WOP; 29.8 % are delivered within the first week after term (from 40+0/7 to 40+6/7 WOP); in the second week after this date, the rate drops to 14.7 %, and only 0.95 % become true postterm pregnancies (>41+6/7 WOP).

**Table 1:** Delivery rate per week of gestation for singleton pregnancies without malformation in BW from 2004 to 2009 for 37+0/7–42+6/7 WOP and beyond

WOP+days	Births in WOP (*N*)	Births in WOP (%)
37+0/7–37+6/7	38,303	8.10
38+0/7–38+6/7	93,447	19.76
39+0/7–39+6/7	126,167	26.68
40+0/7–40+6/7	140,765	29.77
41+0/7–41+6/7	69,663	14.73
42+0/7–42+6/7	4,272	0.90
>42+6/7	226	0.05
Total	472,843	100.00

Table 2 compares the rates of delivery per week of gestation (from 37+0/7 to 41+6/7 WOP) between cohorts reported in the literature [7, 18, 20, 25]. The term births that occurred in Scotland, England and Sweden from 1985 to 1996 were less frequent in weeks 37 and 38 than described in the studies with data from 1997 to 2009 reported from California and BW. During 39 WOP, the rate of deliveries is 4.6–7.7 % points higher in Sweden, California and BW than in Scotland and England. In contrast to this, the delivery rate with 40 weeks is highest in Scotland and England. The birth rate for 41+0/7–41+6/7 WOP is substantially lower in California and BW compared to the older studies from Scotland, England and Sweden (Table 2). It should also be pointed out that in Scotland, England, Sweden, and California, the rate of true postterm pregnancies is above 4 %, while in BW, the rate of true postterm deliveries is only 0.95 %.

**Table 2:** Comparison of delivery rates per week for 37+0/7 to 42+6/7 WOP and beyond, in Scotland [18], England [7], Sweden [20], California [25], and BW

WOP+days	Scotland 1985–1996*N* = 700,878 (%)	England 1989–1991*N* = 158,171 (%)	Sweden 1987–1996*N* = 656,134 (%)	California 1997–2006*N* = 3,820,826 (%)	BW 2004–2009*N* = 472,843 (%)
37+0/7–37+6/7	4.88	5.68	5.37	8.81	8.10
38+0/7–38+6/7	12.73	13.92	14.52	19.13	19.76
39+0/7–39+6/7	21.04	21.07	25.64	28.78	26.68
40+0/7–40+6/7	35.17	34.46	30.03	25.57	29.77
41+0/7–41+6/7	20.88	18.33	17.92	13.31	14.73
42+0/7–42+6/7	5.13	5.34	6.53^a^	4.4	0.90
>42+6/7	0.18	1.19		^b^	0.05
Total	100.00	100.00	100.00	100.00	100.00

^a^Only data given for >41+6/7

^b^No data available

We have calculated the rate of stillbirths based on the number of pregnancies going into a WOP. The numbers of live births and stillbirths, the number of intrauterine pregnancies going into a WOP, and the rate of stillbirths per 1,000 pregnancies going into a WOP in BW are given in Table 3. The risk of stillbirth at 37 WOP is 0.22 per 1,000 ongoing pregnancies. No rise is found at 38 weeks, whereas with 39 weeks the risk is increasing and doubles at 40 weeks. From weeks 40–41, the risk remains approximately constant. The risk of stillbirth increases again when pregnant women enter the true postterm period (>41+6/7 WOP); 95 % CIs are very wide because of the small numbers of IUFD. For comparison, the last column in Table 3 lists the rate of stillbirths per 1,000 births of the week, a parameter which is still calculated in some countries. With that misleading calculation, the levels steadily drop from the 37th week of pregnancy and only start rising again after 41+6/7 WOP.

**Table 3:** Live births, stillbirths, and pregnancies going into the given week, and the rate of stillbirths per 1,000 (based on the number of ongoing pregnancies and births for this week) in BW for the period 2004–2009

Time period: WOP+days	Ongoing pregnancies (at beginning of WOP)	Births in time period			Stillbirths per 1,000 going into the WOP (95 % CI)	Stillbirths per 1,000 births in WOP (95 % CI)
		Live	Dead	Total		
37+0/7–37+6/7	472,843	38,197	106	38,303	0.22 (0.19–0.27)	2.77 (2.29–3.35)
38+0/7–38+6/7	434,540	93,345	102	93,447	0.23 (0.19–0.28)	1.09 (0.90–1.32)
39+0/7–39+6/7	341,093	126,053	114	126,167	0.33 (0.28–0.40)	0.90 (0.75–1.09)
40+0/7–40+6/7	214,926	140,664	101	140,765	0.47 (0.39–0.57)	0.72 (0.59–0.87)
41+0/7–41+6/7	74,161	69,632	31	69,663	0.42 (0.30–0.59)	0.44 (0.31–0.63)
42+0/7–42+6/7	4,498	4,269	3	4,272	0.67 (0.24–1.95)	0.70 (0.26–2.05)
>42+6/7	226	224	2	226	8.85 (2.75–31.60)	8.85 (2.75–31.60)
Total		472,384	459	472,843		

**Fig. 1:**
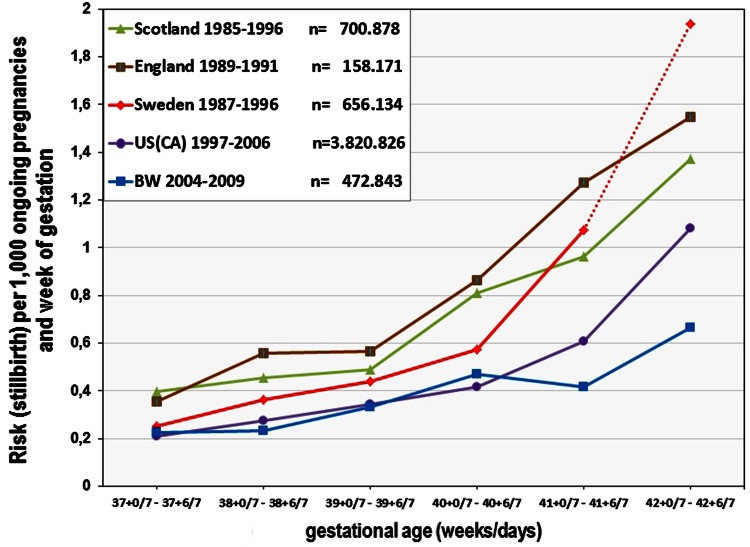
Stillbirths per 1,000 pregnancies going into the WOP for Scotland, England, Sweden, California and BW. The *graph* shows the number of stillbirths per 1,000 pregnancies delivered at or beyond term (37+0/7–42+6/7 WOP) per 1,000 ongoing pregnancies using our data (*blue line*) and the data taken from Hilder [7] (England = *brown line*), Smith [18] (Scotland = *green line*), Divon [20] (Sweden = *red line*, the value for 42+0/7–42+6/7 shows all cases with >41+6/7 WOP), and Rosenstein [25] (California = *violet line*). Comparison of risks for 41+0/7–41+6/7: (Fisher’s exact test): BW—Scotland: *p* < 0.001; BW—England: *p* < 0.001; BW—Sweden: *p* < 0.001; BW—California: *p* < 0.05

For the data published from England [7], Scotland [18], Sweden [20], and California [25], we also calculated the weekly risk of stillbirth per 1,000 ongoing pregnancies (Fig. 1). While the studies from Scotland, England and Sweden show a substantial increase of the fetal death rate, the study from California and our own results are very similar before 41 WOP. For 41+0/7 to 41+6/7 WOP, the risk of stillbirth stays low (0.42/1000 ongoing pregnancies) in BW, while in California it increases to 0.61/1000 (this difference is statistically significant; *p* = 0.046, Fisher’s exact test).

## Discussion

Most guidelines on how to proceed in case of pregnancies over term favor induction of labor at 41+0/7 WOP to prevent unexpected IUFD [9–12]. The recommendations are based on systematic reviews with meta-analysis of data from prospective randomized trials dominated by one randomized multicenter trial from Canada [24]. Additionally, all trials were published 7–37 years ago [15, 22, 23]. Only one of the randomized trials (a study with only 180 and 165 patients in the randomized arms) is from middle Europe and reports a fetal monitoring protocol as practiced in Germany [27]. Both the conclusions and serious limitations of these reviews, therefore, have been discussed controversially [28].

Retrospective analyses of the risk of IUFD in pregnancies at or over term have exclusively been published from English speaking countries, East Asia and Scandinavia [2, 3, 6, 7, 18, 20]. Most of these trials feature a monitoring protocol which cannot be compared with German practice, although to date, the efficacy of fetal monitoring by cardiotocography (CTG) in preventing IUFD in low-risk pregnancies beyond 40+0/7 WOP could not be demonstrated.

For the 472,843 low-risk singleton pregnancies over a period of 6 years, our study demonstrates a markedly lower risk of IUFD compared with the retrospective analyses from Scotland, England, and Sweden. In particular, it should be noted that in BW, the risk level is overall lower and its curve is rather flat until 41+6/7 WOP. The risk only rises after reaching the postterm stage (>41+6/7 WOP). However, it must be considered that the studies from Scotland, England, and Sweden use data, which were collected between 1987 and 1996 and, thus, are 15–20 years older than the data from BW. In addition, the timing of deliveries is significantly different, regarding the elder studies. 54–61 % of the babies were delivered beyond 40+0/7 WOP in Scotland, England, and Sweden, whereas in BW, only 45 % were born after 40+0/7 WOP. This could contribute to the difference found in the rate of IUFD. Therefore, we decided to take the data from California, collected between 1997 and 2006 and published recently [25], for direct comparison with our data. The distribution of the deliveries is similar for California and BW. Both populations show a shift to earlier deliveries with 37, 38 and, particularly marked, with 39 WOP, while the older studies from Scotland, England, and Sweden show higher delivery rates between 40+0/7 and 41+6/7 WOP. The older studies and the study by Rosenstein et al. [25] report a 4–7 % rate of pregnancies delivered beyond 41+6/7 WOP (Tab. 2). We cannot decide whether this is due to a policy of expectant management or due to dating errors as the trials from Scotland [18] and England [7] report no confirmation of the estimated due date by early ultrasound measurement. Additionally, this is also true for the results reported from California [25]. Although the Swedish data pertain to pregnancies with early ultrasound screening [20], the rate of postterm pregnancies (>41+6/7) is still 6.5 %. Therefore, we assume that this is due to a different management policy in Sweden during the study period.

Since German practice uniformly calls for fetal monitoring every 2 days, starting at 40+0/7 weeks of gestation, it is instructive to look more closely at the management policy concerning delivery and fetal monitoring in the different studies. A survey of Swedish departments of obstetrics in 1996 [16], asking for management options and fetal monitoring in postdate pregnancies, revealed that CTG monitoring up to 41+0/7 WOP was performed in just 5 % of cases and only reached 95 % at 42+0/7 WOP and beyond in otherwise normal pregnancies; 87 % of the delivery units would not electively induce labor before 42+0/7 WOP. These different management strategies could explain the significantly lower rates of IUFD in the BW cohort, yet, we cannot decide whether they are caused by the earlier deliveries or by the German monitoring protocol; possibly, both factors may contribute to the different fetal mortality rates, particularly beyond 41+0/7 WOP.

The comparison with the Californian Study [25] shows no striking differences in the fetal mortality rates between 37+0/7 and 40+6/7 WOP; thereafter, the fetal mortality rate is significantly lower in BW. As the distribution of deliveries is not remarkably different between 37 and 41 WOP and as the rate of deliveries is even lower in California between 41+0/7 and 41+6/7 (Table 2), it is a possible assumption that the early starting of fetal monitoring in BW could play an important role. As these are both retrospective cohort studies, there are limitations which must be taken into account: in California, during the years of the study, few low-risk women got antenatal testing at 40 weeks, with potentially more tests starting at 41 weeks if they declined induction (Rosenstein MG, personal communication), while in BW the serial monitoring between 40+0/7 and 41+6/7 WOP has been standard since the 1990s in all obstetric units (Weiss E: survey BW 2012, unpublished).

On the other hand, in BW as well as in all compared populations, the risk of IUFD beyond 41+6/7 WOP increases rather steeply (Fig. 1) despite fetal monitoring, which is recommended in all published guidelines for pregnancies continuing after 41+6/7 WOP [9–12]. However, in BW the risk of IUFD beyond 41+6/7 WOP is markedly lower than in all studies used for comparison. Their considerably higher fetal mortality beyond 41+6/7 WOP is particularly interesting because in these countries the percentage of pregnancies going beyond 41+6/7 WOP is high. It is not clear whether the differences in accurate pregnancy dating by routine first trimester ultrasound contribute to this results. A possible—but in a retrospective study not provable—explanation could be that the serial monitoring between 40+0/7 and 41+6/7 WOP in BW is able to identify cases with low placental reserve capacity. Consequently, this may lead to a medically indicated induction of labor before these pregnancies enter the dangerous postterm period (> 41+6/7 WOP).
